# Autonomic dysfunction is common in liver cirrhosis and is associated with cardiac dysfunction and mortality: prospective observational study

**DOI:** 10.1590/1516-3180.2021.0111.R1.18052021

**Published:** 2021-11-29

**Authors:** Carolina Frade Magalhães Girardin Pimentel, Ricardo Salvadori, Ana Cristina de Castro Amaral Feldner, Miguel Osman de Aguiar, Adriano Miziara Gonzalez, Gabriel Ribas Branco, Marcel Superbia, Michelle Lai, Daniel de Oliveira Mota, Maria Lucia Cardoso Gomes Ferraz, Wilson Mathias, Mario Kondo

**Affiliations:** I MD, PhD. Professor, Department of Medicine, Universidade Federal de São Paulo (UNIFESP), São Paulo (SP), Brazil.; II MD. Physician, Department of Electrophysiology, Hospital São Luiz and Hospital e Maternidade São Luiz, Unidade Itaim, São Paulo (SP), Brazil.; III MD, PhD. Physician, Department of Gastroenterology, Universidade Federal de São Paulo (UNIFESP), São Paulo (SP), Brazil.; IV MD. Physician, Department of Echocardiology, Hospital e Maternidade São Luiz, Unidade Itaim, São Paulo (SP), Brazil.; V MD, PhD. Professor, Department of Surgery, Universidade Federal de São Paulo (UNIFESP), São Paulo (SP), Brazil.; VI MD. Physician, Department of Echocardiology, Hospital e Maternidade São Luiz, Unidade Itaim, São Paulo (SP), Brazil.; VII MD. Physician, Department of Echocardiology, Hospital e Maternidade São Luiz, Unidade Itaim, São Paulo (SP), Brazil.; VIII MD. Physician, Liver Center, Beth Israel Deaconess Medical Center, Harvard Medical School, Boston, MA, United States.; IX PhD. Engineer and Professor, Department of Industrial Engineering, Universidade de São (USP), São Paulo (SP), Brazil.; X MD, PhD. Professor, Department of Gastroenterology, Universidade Federal de São Paulo (UNIFESP), São Paulo (SP), Brazil; and Research Coordinator, Instituto D’Or de Pesquisa e Ensino (IDOR), São Paulo (SP), Brazil.; XI MD, PhD. Professor, Department of Cardiology, Instituto do Coração (InCor), Hospital das Clinicas HCFMUSP, Faculdade de Medicina, Universidade de Sao Paulo, Sao Paulo, SP, BR.; XII MD, PhD. Professor, Department of Gastroenterology, Universidade Federal de São Paulo (UNIFESP), São Paulo (SP), Brazil.

**Keywords:** Liver cirrhosis, Heart rate, Mortality, Heart diseases, Autonomic dysfunction, Cirrhotic cardiomyopathy, Heart rate variability

## Abstract

**BACKGROUND:**

Although autonomic dysfunction has been shown to be associated with liver cirrhosis, the prevalence and prognostic implications are unclear. Abnormal heart rate variability (HRV), a measure of autonomic function, has not been well investigated in cirrhosis.

**OBJECTIVE:**

To evaluate the prevalence of high-risk HRV parameters in a cohort of cirrhotic patients and their association with cardiac dysfunction and mortality.

**DESIGN AND SETTING:**

Prospective observational study conducted in the Federal University of São Paulo.

**METHOD:**

A cohort of 120 patients, comprising 17 healthy controls and 103 cirrhotic outpatients, was evaluated and followed for 10 months. HRV analysis was based on 24-hour Holter monitoring and defined using time-domain and frequency-domain parameters.

**RESULTS:**

The HRV parameters were statistically lower in cirrhotic patients than in healthy subjects. High-risk HRV parameters were prevalent, such that 64% had at least one high-risk parameter. Time-domain parameters correlated with Child scores (P < 0.0001). In regression models, HRV parameters were independent predictors of diastolic dysfunction and mortality. During 10 months of follow-up, there were 11 deaths, all of patients with at least one high-risk HRV parameter. Kaplan-Meier analysis estimated low survival rates among patients with standard deviation of normal-to-normal RR intervals (SDNN) < 100.

**CONCLUSION:**

Reduced HRV is prevalent in liver cirrhosis and is related to cardiac dysfunction, severity of liver disease and mortality. Abnormal high-risk HRV parameters are prevalent among cirrhotic patients and are also predictors of mortality. Our findings highlight the need for a more careful cardiac evaluation of cirrhotic patients.

## INTRODUCTION

Liver cirrhosis has a wide spectrum of clinical extrahepatic organ manifestations. The heart is affected in the majority of patients, ranging from mild to severe impairment. The latter is more prevalent during end-stage liver disease.^1^ Cirrhosis with portal hypertension is characterized by circulatory dysfunction with chronic peripheral vasodilation, leading to insufficient organ perfusion and a compensatory increase in cardiac output.^2^ This hyperdynamic circulation requires an increase in heart rate (HR), which is dependent on the autonomic nervous system (ANS). Dysfunction of the ANS leads to an abnormal HR response, especially in situations when peripheral oxygen demand increases, as during hemodynamic stress or exercise.^3,4^

QT interval prolongation has been shown to correlate with cirrhotic cardiomyopathy,^1^ representing one manifestation of electrophysiological abnormalities and autonomic dysfunction. However, few studies have demonstrated the relationship between these electrocardiographic abnormalities and outcomes in liver cirrhosis.^5,6,7^

Another way to access cardiac autonomic function is to measure heart rate variability (HRV), which involves analysis of consecutive normal R-R intervals (also called N-N) during a period of time. HRV reflects the heart’s ability to adapt HR to changing circumstances by detecting and quickly responding to unpredictable and variable stimuli.^8^ Sympathetic and parasympathetic system impairment leads to improper acceleration or insufficient increase in HR. A number of different measurements are used to analyze HRV during 24-hour Holter monitoring; these parameters are classified into two distinct groups: time-domain and frequency- domain parameters. Time-domain parameters are most frequently used to diagnosis reduced HRV, while frequency-domain analysis estimates the amount and type of variance in this reduction, thus characterizing whether the autonomic dysfunction is due to an increased sympathetic tone or to parasympathetic impairment.

Autonomic dysfunction is considered to be a feature of liver cirrhosis, presented as autonomic imbalance secondary to relatively decreased parasympathetic activity and increased sympathetic tone.^9,10^ Different factors seem to contribute to this dysfunction in cirrhosis, such as direct nerve injuries due to alcohol toxicity, alteration of lipid metabolism, vitamin E deficiency, immunological mechanisms and higher toxic metabolite concentrations.^10,11^ In addition, elevated angiotensin II levels affect vagal function and elevated nitric oxide levels reduce the vascular response to norepinephrine. Cirrhotic patients with vagal neuropathy have fivefold higher mortality than those without this.^10,12^

The main use of HRV analysis has been in risk-stratifying patients with regard to malignant arrhythmias and death after myocardial infarction (MI).^8,13,14^ Small studies have demonstrated that decreased HRV is present in liver cirrhosis, and that it may be an indicator of poor prognosis and mortality,^5,15,16^ Although autonomic dysfunction is a known complication in this group, these previous studies using HRV to diagnose autonomic dysfunction were based on short-time analysis, rather than continuous monitoring using 24 hour Holter electrocardiograms.^12,17,18^ In addition, several of these study populations comprised patients with well-compensated liver disease, so the prevalence of autonomic dysfunction found were not reflective of the prevalence among patients with more advanced liver disease. While post-MI patients with ANS impairment have been found to be at higher risk of sudden cardiac death and malignant arrhythmias, it has not been demonstrated whether ANS impairment also confer the same risks among patients with cirrhosis.^19^ There is no study in the literature comparing the relationship between decreased HRV and features of cirrhotic cardiomyopathy. Moreover, it is unclear whether autonomic cardiac dysfunction is the primary event or a consequence of cardiovascular dysfunction in liver cirrhosis.

## OBJECTIVE

The aim of this study was to evaluated a large cohort of cirrhotic patients in order to detect the prevalence of autonomic dysfunction, as represented by decreased HRV and QT prolongation, and its relationships with advanced liver disease, clinical decompensation, mortality and underlying cardiac dysfunction.

## METHODS

### Patients and methods

A total of 120 subjects, comprising 103 outpatients with liver cirrhosis (57 male; 46 female; mean age 51.2 ± 12.9 years) and 17 healthy controls (6 male; 11 female; mean age 50 ± 13.4 years) were included in the present study. Cirrhosis was defined from the subjects’ clinical histories (alcohol abuse, hepatitis infection, genetic disorder or metabolic syndrome), physical examinations, laboratory analyses (low albumin, high bilirubin, high prothrombin time and thrombocytopenia) and data from at least one imaging examination (nodularity and increased echogenicity, atrophy of the right lobe and hypertrophy of the left one, portal vein enlargement and splenomegaly). All patients underwent blood sample collection within one week of Holter analysis, in order to exclude patients with significant electrolyte disturbances. The exclusion criteria consisted of any previous cardiovascular disease, heart failure or diagnosis of hemochromatosis. Patients who had a history of alcohol abuse (more than 20 g of ethanol per day for women and more than 60 g for men)^20^ would need to have abstained for at least six months prior to enrollment. Patients who presented a recent history (less than three months) of liver-related decompensation or hospitalizations were also excluded. Healthy controls were included if there was no previous diagnosis of liver disease and their echocardiograms showed normal systolic and diastolic functions.

A total of 164 patients at two liver transplantation centers were consecutively screened between October 2014 and December 2014. Among these, 61 were excluded based on the criteria listed above. The patients were followed up through clinical visits, hospital records or telephone calls to them. They were asked to provide their written informed consent on the day of enrollment. All patients underwent laboratory tests, electrocardiograms (ECG), transthoracic echocardiograms (ECO) and 24-hour Holter monitoring within one month of enrollment. This study was conducted in accordance with the Declaration of Helsinki (2000) and was approved by the ethics committee of our institution (CAAE: 30942714.8.0000.5505; dated: May 28, 2014).

### QT interval and Holter monitoring

The QT interval (QT) was corrected for heart rate (QTc) using the Bazzett formula^21^ and considered prolonged if greater than 440 ms. 24-hour Holter monitoring was obtained using a portable battery-operated three-channel Cardio-Light recorder and was processed using the Cardio Smart S-550 Cardio Sistemas Holter analysis software (CardioSmart, São Paulo, Brazil). After automatic QRS detection, the data were reviewed by an experienced Holter analyst. Patients without sinus rhythm were excluded. In the rhythm analysis, all parameters were calculated per hour, and were presented as 24-hour means for statistical analysis.

Abnormal HRV was analyzed as a marker of autonomic dysfunction and chronotropic incompetence, in accordance with current guideline recommendations.^22^ Time and frequency-domain parameters were calculated. The time domain analyses were reported as follows: the standard deviation of normal-to-normal RR intervals (SDNN-ms), representing overall autonomic dysfunction; the mean of the standard deviations of all NN intervals for all five- minute segments of the entire recording (SDNNIX); the standard deviation of the average N-N interval for each five-minute period over 24 hours (SDANN-ms), representing the sympathetic component of autonomic function; the square root of the mean of the squared differences between adjacent NN intervals (rMSSD-ms), which was correlated with parasympathetic activity; and the percentage of adjacent NN intervals that were > 50 msec (pNN50%), which was also correlated with the parasympathetic component. The data of the frequency domain were represented by total power (TP ≤ 0.4 Hz); very low-frequency power (VLF: 0.0033-0.04 Hz), which might represent the influence of the thermoregulatory, peripheral vasomotor or renin-angiotensin systems; low-frequency power (LF: 0.04-0.15 Hz), modulated by the sympathetic system; high-frequency power (HF: 0.15-0.49 Hz), modulated by the parasympathetic system; and the LF/HF ratio, which reflected the sympathetic-vagal balance.^22^ The VLF, LF and HF components were expressed in ms^2^ or nu (normalized units). The normal cutoff values described in the literature were considered to be standards.^22^ The cutoffs for defining abnormal parameters associated with high risks of unfavorable outcomes (malignant arrhythmias and sudden cardiac deaths) were based on the European guidelines and on post-MI studies in the heart failure population.^13,22^ The values reported in the literature are SDNN < 100 ms, 70 ms or 50 ms; SDANN < 100 ms or 55 ms; rMSSD < 15 ms; and pNN50 > 5%.^14^

### Echocardiograms

Transthoracic echocardiogram studies was performed using Vivid E9 with the M5S transthoracic transducer from General Electric Medical Systems, Milwaukee, Wisconsin, United States. Left ventricular (LV) systolic function was represented by the ejection fraction (EF) (Simpson’s biplanar method) and longitudinal global strain. Systolic dysfunction was defined as EF ≤ 55% or strain ≥ -18%. LV diastolic function was evaluated based on early and late peak diastolic velocities (E/A; estimated through Doppler analysis and considered normal if more than 1.0); the ratio between early diastolic transmitral flow velocity and lateral mitral annulus motion (E/e’; normal if less than 8.0);^23^ and the deceleration time (DT; abnormal if greater than 240 ms). E/e’ was considered to be the reference for diastolic dysfunction, given that this ratio is less influenced by preload and cardiac afterload.

### Statistical analyses

The data were analyzed using a statistical software program (IBM SPSS Statistics, version 22.0) (IBM, Armonk, United States). Two-tailed Student’s t tests and analysis of variance (ANOVA) were calculated for pairwise comparisons of continuous variables. Post-hoc testing was performed by using the Games-Howell test. Pearson’s correlation test was performed to compare continuous variables. Categorical variables were expressed as percentages and analyzed using the chi-square test or Fisher’s exact test, as applicable. Linear and logistic regressions were performed to evaluate associations between independent variables, HRV parameters or QTc intervals, and the presence of cardiac dysfunction and death. Receiver operating curves (ROC) and the area under this curve (AUROC) were computed to estimate sensitivity, specificity and cutoff points for the HRV parameters used in regression models. Kaplan-Meier curves were built and significant differences between them were assessed by means of the log-rank test. Statistical significance was considered present when P < 0.05, using two-tailed tests.

## RESULTS

### Patients’ characteristics

The main demographic, clinical and laboratory characteristics of the patients are presented in Table 1. The healthy control patients had a mean age of 50 ± 3.8 years, and no statistical difference was found between this group and the cirrhotic patients. A total of 106 patients were selected from two liver transplantation centers in São Paulo, Brazil. The majority of them were male (56%), and the etiology of the liver disease was most commonly non-alcoholic (69.8%). The mean model for end-stage liver disease (MELD) was 11.1, Child B was most common (66%) and 74% of the patients presented a history of at least one liver-related decompensation. Ascites was identified in 32.1% and hepatic encephalopathy in 10.4% of the patients on the day of the test.

**Table 1.** Patients’ characteristics

Characteristic	
**Gender M/F, n (%)**	57/46 (55.3/44.7)
**Age (years ± SD)**	51 ± 13
**Cirrhosis etiology, n (%)**	
Virus	33 (35)
Alcohol	32 (31.1)
NASH	8 (7.8)
Others	30 (29)
**Child-Pugh (mean ± SD)** **Child-Pugh class, n (%)**	7.1 ± 1.8
A	23 (22.3)
B	68 (66)
C	12 (11.7)
**MELD (mean ± SD)**	11.1 ± 3.1
**History of liver-related decompensation, n (%)**	76 (73.8)
**Hypertension, n (%)**	19 (18.4)
**Diabetes, n (%)**	16 (15.5)
**Beta-blocker in use, n (%)**	34 (30.1)
**Baseline laboratory tests (mean ± SD)**	
Hemoglobin (mg/dl)	13.1 ± 1.9
Na (mmol/l)	137.8 ± 2.1
K (mmol/l)	4.1 ± 0.5
Mg (mg/dl)	1.8 ± 0.2
Ca^2+^ (mmol/l)	1.2 ± 0.1

Reference range values: Na (136-145); K (3.5-5.0); Mg (1.6-2.6) and Ca^2+^ (1.15-1.29). M/F = male/female; NASH = nonalcoholic steatohepatitis; MELD = model for end-stage liver disease; Na = sodium; K = potassium; Mg = magnesium; Ca = calcium.

All the patients were followed until death, time of transplantation or end of study follow-up (10 months). During the study period, 11 patients died and three underwent liver transplantation.

### Echocardiograms

All the healthy patients presented normal echocardiographic values. More than half of this cohort (57%) had one or more features of diastolic dysfunction: 24% with E/e’ > 8 (n = 25), 8.5% with E/A < 0.8 (n = 9) and 37% with DT > 240 ms (n = 38). Only 5 patients presented features of systolic dysfunction, as represented by EF ≤ 55% or strain ≥ -18%. The echocardiographic parameters, except for LA, did not differ between Child groups. Although 42% of Child C patients presented E/e’ > 8.0, compared with 28% of Child A and B patients (n = 20), there were no statistically significant differences among Child groups (P = 0.18).

### HRV and QTc analyses

QTc means were significant higher in cirrhotic patients (445 ± 29 ms) than in healthy controls (429 ± 19 ms) (P = 0.04). A prolonged QTc interval was frequent in this population (60.2%) and progressively longer among Child classes: Child A (428 ± 35 ms), Child B (449 ± 26 ms) and Child C (451 ± 25 ms). Statistical differences were demonstrated between Child A and B (P = 0.003) and between Child A and C (P = 0.02). Student’s t test analyses did not show any differences between QTc means according to cardiac dysfunction, history of liver-related decompensations, alcohol consumption, use of beta blockers or death (P > 0.05). No electrolyte disturbance or anemia was present at the time of the evaluation.

**Figure 1.**
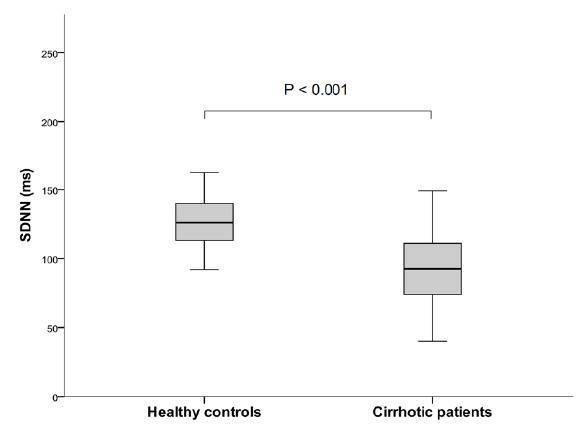
SDNN means are higher in the control group than in the cirrhotic patients.

Reduced HRV was frequently detected among cirrhotic patients. There were statistical differences in time-domain parameters, including SDNN, SDANN, SDNNIDX and rMSSD, between cirrhotic patients and healthy controls (Figure 1). In addition, TP, VLF and LF means were significant higher in controls than in cirrhotic patients (Tables 2 and 3).

Mortality associated with SDNN less than 104 ms.

**Table 2.** Heart rate variability (HRV) parameters

Parameter	Controls	Cirrhotic patients	P^*^
NN (ms)	810 ± 88	793 ± 110	0.27
SDNN (ms)	130 ± 29	94.8 ± 31	< 0.001
SDANN (ms)	110 ± 26	82.7 ± 29	< 0.001
SDNNIDX (ms)	60 ± 20	40.6 ± 16	< 0.001
rMSSD (ms)	36 ± 25	25.0 ± 13	0.051
pNN50 (%)	9.1 ± 9.7	4.4 ± 6	0.121
TP (ms^2^)	2823 ± 2250	1438 ± 1611	0.002
VLF (ms^2^)	1338 ± 932	823 ± 909	0.033
LF (ms^2^)	969 ± 710	411 ± 539	< 0.001
HF (ms^2^)	517 ± 779	206 ± 244	0.122
LF/HF ratio	3.7 ± 1.5	3.8 ± 2.4	0.991

*P (t tests comparing means between control and cirrhotic patients). NN = normal-to-normal; SDNN = standard deviation of normal-to-normal RR intervals; SDANN = standard deviation of the average of N-N intervals for each 5-minute period over 24 hours; SDNNIDX = the standard deviations of all NN intervals for all 5-minute segments of the entire recording; rMSSD = root mean square of successive differences between normal heartbeats; pNN50 = proportion of NN50 divided by the total number of NN (R-R) intervals; TP = total power; VLF = very low-frequency power; LF = low-frequency power; HF = high-frequency power.

**Table 3.** Prevalence of abnormal high-risk heart rate variability parameters and QTc prolongation in cirrhotic and control groups

Parameter	Cirrhotic patients	Controls	P
SDNN < 100 ms	60%	12%	< 0.001
SDNN < 70 ms	20%	0%	0.04
SDNN < 50 ms	9%	0%	0.35
SDANN < 100 ms	77%	29%	< 0.001
SDANN < 55 ms	17%	0%	0.13
rMSSD < 15 ms	24%	6%	0.12
pNN50 > 5%	25%	60%	0.006
QTc > 440 ms	60%	0%	< 0.001

SDNN = standard deviation of normal-to-normal RR intervals; SDANN = standard deviation of the average of N-N intervals for each 5-minute period over 24 hours; rMSSD = root mean square of successive differences between normal heartbeats; pNN50 = proportion of NN50 divided by the total number of NN (R-R) intervals; QTc = QT interval corrected for heart rate.

Reduced HRV parameters were correlated with greater severity of liver disease. The time domain variables SDNN and SDANN correlated with Child scores (P < 0.0001; r = -0.406 and -0.407, respectively). In addition, the means were statistically different among Child stages (P = 0.002 for both), with a tendency to decrease as Child scores increased (for SDNN, Child A = 111 ms, Child B = 93 ms and Child C = 73 ms; P = 0.002). A weak negative correlation between Child scores and TP was also demonstrated (P = 0.033; r = -0.229). HRV parameters were also associated with MELD, albeit poorly correlated (SDNN, P = 0.008, r = -0.261; SDANN, P = 0.005, r = -0.277).

The majority of the cirrhotic patients presented with SDNN less than 100 ms (60%), while just 11.8% of the controls did (P = 0.002). Table 4 shows the frequency of abnormal high risk HRV parameters, in accordance with the European guidelines^22^ and with post-MI studies.^13^ As demonstrated, several parameters were statistically different between the groups: parasympathetic impairment was noticeable through pNN50% > 5%, which was less frequent in cirrhosis; and through reduced rMSSD, which was combined with sympathetic impairment characterized by lower SDANN and LF in cirrhosis.

**Table 4.** Logistic regression model for prediction of diastolic dysfunction and mortality using time and frequency-domain parameters

Predictor	Diastolic dysfunction			Mortality		
	b	P	CI for b	b	P	CI for b
SDNN	−0.31	0.005	−0.047/−0.009	−0.31	0.019	−0.071/−0.008
SDANN	−0.026	0.004	−0.049/−0.009	−0.36	0.01	−0.074/−0.012
rMSSD	−0.007	0.683	−0.052/0.029	−0.014	0.629	−0.012/−0.126
HF(nu)	−0.051	0.004	0.019/0.091	0.033	0.190	−0.027/0.092
LF(nu)	−0.051	0.002	−0.094/−0.016	−0.31	0.210	0.033/−0.091
LF/HF	−0.402	0.007	−0.821/−0.148	−0.291	0.072	−0.832/−0.026

b = value of the model equation coefficient. CI = confidence interval; SDNN = standard deviation of normal-to-normal RR intervals; SDANN = standard deviation of the average of N-N intervals for each 5-minute period over 24 hours; rMSSD = root mean square of successive differences between normal heartbeats; HF = high-frequency power; LF = low-frequency power.

No significant difference in HRV parameters was observed between patients with alcohol-related cirrhosis and those with other etiologies. The t test did not demonstrate any significant difference between these groups, according to the means for SDNN, SDANN, rMSSD, pNN > 50% and QTc.

HRV parameters were associated with Child classes independently of the presence of diabetes, hypertension or use of beta blockers. We analyzed the means for HRV parameters according to Child stages, using one-way ANOVA, in three different subgroups: 1) patients without diabetes; 2) those without hypertension; and 3) those not using beta blockers. We demonstrated that the presence of these factors did not affect the association between cirrhosis and autonomic dysfunction, as we found the same significant difference between classes as previously reported for the entire cohort (P < 0.001, P = 0.001 and P = 0.007, respectively). Therefore, the relationship between HRV parameters and Child groups was unaffected by the association of comorbidities or alcohol consumption in our study.

### Autonomic dysfunction and cardiac dysfunction

**Figure 2.**
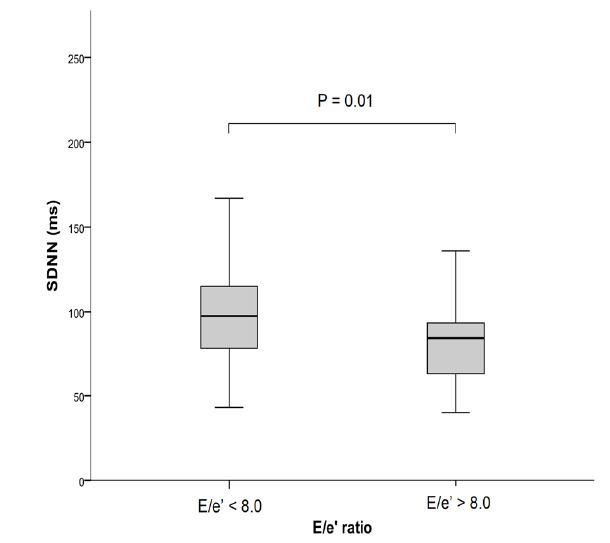
Lower SDNN means in patients with features of diastolic dysfunction, assessed by means of E/e’ ratio.

In cirrhotic patients, reduced HRV was more prevalent in those with features of diastolic dysfunction. The time-domain parameters SDNN and SDANN were significantly lower in patients with E/e’ greater than 8.0 (81 ms versus 99 ms, P = 0.01; and 24 ms versus 78 ms, P = 0.01, respectively) (Figure 2). In addition, HF, LF and LF/HF ratio were significantly different between patients with or without diastolic dysfunction (59 versus 68 nu, 40 versus 32 nu and 2.6 versus 4.1; P = 0.005, P = 0.005 and P = 0.001, respectively).

SDNN = standard deviation of normal-to-normal RR intervals; E/e’ = ratio between early diastolic transmitral flow velocity and lateral mitral annulus motion.

Logistic regression was carried out to assess autonomic dysfunction parameters as predictors of diastolic cardiac dysfunction and mortality in our population (Table 4). Several time-domain and frequency-domain parameters were independent predictors of diastolic dysfunction. In addition, SDNN and SDANN were also reported as independent predictors of mortality according to the model. Also, we analyzed whether reduced HRV was associated with diastolic dysfunction and mortality. SDNN less than 100 ms and LF/HF more than 2 were considered to be independent variables for prediction of diastolic dysfunction (P = 0.018, b = 1.81, confidence interval (CI) for b = 0.213 to 2.57; and P = 0.002, b = -1.52, CI for b = -2.62 to -0.56, respectively). Regarding prediction of mortality, SDNN < 100 ms (P = 0.04, b = -1.94, CI for b = -20.0 to -0.36) and SDANN < 100 ms (P = 0.001, b = 19.25, CI for b = -19.86 to -18.3) were also related to deaths, while LF/HF ratio was not (P = 0.689).

**Figure 3.**
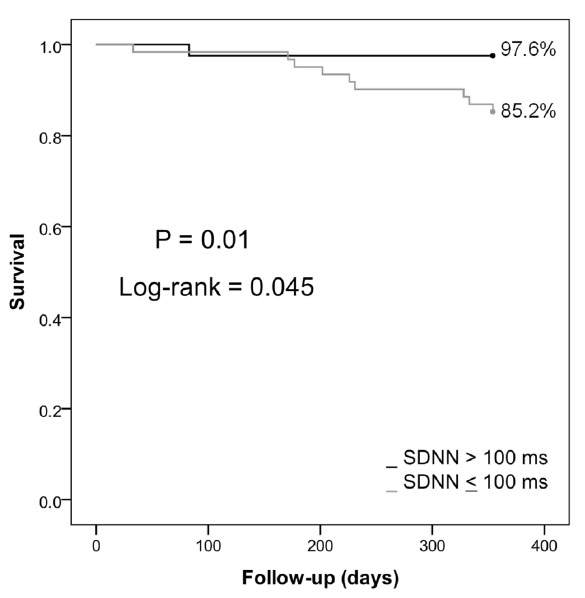
Mortality associated with SDNN less than 104 ms.

The predicted values from the regression model using SDNN were used to build the ROC curve and calculate the AUROC. For prediction of diastolic dysfunction, AUROC was 0.68 (P = 0.01, CI = 0.56-0.79) and for mortality, AUROC = 0.705 (P = 0.034, CI = 0.550-0.859). Sensitivity and specificity, computed using the Youden index, were 91.7% and 43.6% (for diastolic dysfunction) and 100% and 40% (for mortality). The SDNN cutoffs associated with diastolic dysfunction diagnosis and mortality in this model were 106 ms and 72 ms, respectively. The patients who died (11; 10.4%) had at least one high-risk HRV parameter. Ten patients had SDNN less than 100 ms (85.7%), while just one patient did not (SDNN = 103 ms). Kaplan-Meier survival analysis estimated significantly lower survival rates among patients with SDNN < 100 than among those with SDNN ≥ 100 (log-rank, P = 0.045) (Figure 3).

SDNN = standard deviation of normal-to-normal RR intervals.

## DISCUSSION

Autonomic dysfunction is a common finding among cirrhotic patients, documented through either QTc prolongation or reduced HRV.^5,18,24^ This study hereby confirms these findings from previous study, and shows an association of QTc prolongation and HRV with increased mortality. We also found increasing prevalence and severity of autonomic dysfunction with increasing severity of liver disease, regardless of etiology, alcohol consumption or comorbidities. Moreover, this highlights the relationship between autonomic and cardiac dysfunctions, showing high prevalence of ANS impairment in patients with diastolic dysfunction. This relationship gives rise to potential use as a promising tool for cardiac evaluation in liver cirrhosis, as this is more likely to reflect the early changes of cirrhotic cardiomyopathy than are traditional echocardiographic measurements.

Patients with cirrhosis and portal hypertension have a progressively hyperdynamic circulatory state as liver function declines.^2^ Maintenance of adequate peripheral organ perfusion depends on an increase in cardiac output, mainly through HR response to hemodynamic stress.^25^ ANS is a key element in this delicate balance: when this does not work properly, the result is circulatory failure.

Previous studies have demonstrated common vagal neuropathy in cirrhosis, leading to progressive autonomic dysfunction due to parasympathetic impairment.^10,26^ However, these were small studies,^12,27^ mainly among patients without advanced liver disease,^17^ and they were frequently related to alcohol consumption,^28^ which itself causes cardiomyopathy. The study population characteristics of those studies prevented accurate evaluation of the real prevalence of this disorder in cirrhosis. Some studies based this diagnosis on peripheral neuropathy or on tests requiring patients’ compliance (i.e. deep breathing and standing),^9,11,12^ which may result in error if the patient is unable to perform the test correctly. In addition, given that ANS impairment is variable, presenting different patterns during the day and night,^29^ use of 24-hour Holter monitoring seems to be more appropriate for assessing ANS impairment.

Our data from 24-hour Holter monitoring of a large cohort of 103 patients demonstrated a clear association between autonomic dysfunction (reduced HRV) and liver function severity (Child and MELD scores), regardless of previous alcohol consumption. Our cohort of patients had advanced liver disease, and the vast majority (almost 75%) had had at least one episode of liver-related clinical decompensation in the past (ascites, hepatic encephalopathy, variceal bleeding or hepatorenal syndrome). This is the biggest cohort of cirrhotic patients evaluated with regard to ANS impairment using HRV analysis that we are aware of.

The pathophysiology of heart failure in liver cirrhosis is not completely understood.^1^ However, previous studies have suggested that decreased density of beta-adrenoceptors in cardiomyocytes may occur.^30^ This could be responsible for the myocardial hyporesponsiveness to catecholamine, with progressive damage to myocardial tissue, thus leading to cardiac pump dysfunction. The hyperdynamic circulation that defines cirrhotic cardiomyopathy does not present with traditional echocardiographic features of diastolic and systolic cardiac impairment until late in the disease process. In our cirrhosis cohort, we found that ANS imbalance was: 1) more prevalent than cardiac dysfunction; 2) associated with liver disease severity; and 3) predicted cardiac dysfunction. We suspect that ANS disorders may be the first event in cirrhotic cardiomyopathy. This suggests that screening for cirrhotic cardiomyopathy through testing for autonomic dysfunction would lead to earlier diagnosis, thus enabling possible earlier intervention or risk stratification for liver transplantation candidates. Further studies to test this specific hypothesis are required.

Although QTc is commonly prolonged in cirrhotic patients, we did not demonstrate any relationship between this parameter and cardiac dysfunction. Also, Qtc > 440 ms was more prevalent in the later stages of liver disease, but we could not identify any statistical association between this parameter and mortality risk. Genovesi et al.^5^ demonstrated a significant correlation for QTc and hepatic vein pressure gradient (HVPG) in relation to Child scores, but no association with survival was demonstrated. Bernardi et al. suggested that QTc prolongation might have an important prognostic meaning,^31^ but we, along with other studies, were unable to prove this association.^7^ The high prevalence of QTc prolongation is clinically important in this patient population, in which some commonly used drugs (ciprofloxacin and furosemide) or associated electrolyte disturbances may increase the risk of arrhythmias by additionally prolonging the QT interval.

Although QTs is described as more frequently prolonged in patients with a history of alcohol intake who are not using beta-blockers,^32^ we did not find any statistical differences based on histories of either alcohol use or beta-blocker use. Patients taking beta-blockers are likely to have more advanced disease (requiring prophylaxis for primary or secondary variceal bleeding). Because beta-blockers can decrease the QTc, the actual untreated mean QTc in this group of patients may be longer than what was observed. In the present study, reduced HRV was frequently detected in cirrhotic patients; also, time and frequency-domain parameters were significant lower in this group than in the healthy subjects. Our data confirm the results of previous studies that indicated a lack of cardiac chronotropic response in cirrhosis (reduced SDNN), as a consequence of parasympathetic impairment (reduced rMSSD) and sympathovagal imbalance (reduced LF/HF ratio).^12,15^ Unlike the data in the literature, we also identified reduced sympathetic tone in our cohort (reduce SDANN and low LF). With the availability of modern electrocardiographic recorders and new software, 24-hour Holter monitoring enables a more comprehensive assessment of the pathophysiological pathways involved in autonomic dysfunction, and may help identify future treatment targets: for example, exercise training, which has previously been correlated with increased HRV in experimental studies.^33^

Baratta et al.^27^ studied 30 cirrhotic patients waiting for liver transplants and did not find any relationship between cirrhosis severity (MELD) and the degree of autonomic dysfunction. On the contrary, in 30 another subjects, Ates et al.^15^ demonstrated that there was a significant correlation between Child scores and time-domain parameters. The diversity of results found in the literature may be explained by the use of small cohorts, and by differing severities of liver disease in different subject populations. Here, we reported that there was quite a strong inverse correlation between SDNN and Child scores (r = -0.4; P < 0.0001) and a weak one with MELD (r = -0.3; P = 0.008), thus indicating more overall autonomic dysfunction with worsening liver function. The relationships between HRV parameters and Child groups were unaffected by the association of comorbidities (hypertension and diabetes) or alcohol intake, thus suggesting that liver function has a predominant influence on autonomic dysfunction.

In 1987, Kleiger et al. demonstrated that, post-MI, reduced HRV (defined as SDNN < 50 ms) was highly associated with sudden cardiac death and malignant arrhythmias.^13^ Rovere et al. published the ATRAMI Study results, in which preserved HRV variability, defined as SDNN > 105 ms, gave rise to lower mortality rates than among patients with reduced HRV, thus reinforcing the value of HRV as a prognostic factor.^34^ The European guidelines for HRV assessment define the cutoff value for highly depressed HRV as SDNN as < 50 ms, and for moderately depressed HRV as SDNN < 100 ms. In addition, no currently recognized HRV measurements provide better prognostic information, post-MI, than time-domain HRV measurements, in special SDNN and HRV triangular indexes.^22^ Based on this, we decided to focus on SDNN analysis in our cohort.

Our data from this large cohort confirm the finding from some previous small studies that reduced HRV is an independent mortality risk factor in cirrhosis.^10,12,15^ During the ten-month follow-up, 11 patients died, with lower SDNN means (75 ± 24 ms versus 96.9 ± 31.2 ms) and lower SDANN (64 ± 18.7 ms versus 84.8 ± 28.8 ms). We reported that were significant differences in Kaplan-Meier survival curves according to SDNN values (P = 0.045), and it was noticeable that longer follow-ups may better highlight this difference.

This is the first study in the literature to investigate the association between reduced HRV and cardiac dysfunction. We identified that 60% of our patients presented with SDNN < 100 ms, thus drawing attention to the arrhythmia risk in this population. Moreover, we reported that there was an independent association with SDNN for prediction of diastolic dysfunction when it was higher than 105 ms. We hypothesize that ANS disorder is the first event in cirrhotic cardiomyopathy, such that this would require careful attention during cirrhosis management. Currently, liver transplant evaluations rely on transthoracic echocardiograms to diagnose cardiac dysfunction. Most of the current protocols do not routinely include autonomic dysfunction evaluation and therefore they may potentially miss the diagnosis of reduced HRV, which predisposes patients to poor outcomes during hemodynamic stress, as occurs during liver transplantation.

One potential limitation of the present study is that we did not exclude patients with diabetes, which is a known risk factor for autonomic dysfunction. For a more reliable interpretation, we carried out individual analyses comparing patients with and without this disorder, thus preventing bias in the interpretation, and no further difference between groups was detected. Another point to be noted is our inclusion of cases of alcoholic cirrhosis. However, all patients had been abstinent for at least six months, which will have decreased the direct alcohol neuropathy. We also compared patients with and without histories of alcohol intake, and the diagnoses of autonomic dysfunction were similar in both groups. Another weakness is that our evaluation was conducted among outpatients, although it is known that cardiac dysfunction in cirrhosis is more prone to be manifested during hemodynamic stress. Future protocols should be developed to evaluate cardiac function during cirrhosis decompensation; however, several confounders may prevent determination of causality related to liver disorders. Lastly, although our control group had more women than men, this difference was not statistically significant (P = 0.19). Moreover, no HRV differences between genders have been reported in the literature.

## CONCLUSIONS

Our study shows that reduced HRV is prevalent in liver cirrhosis, and that this is related to cardiac dysfunction, severity of liver disease and mortality. We also report that abnormal high-risk HRV parameters, which had previously been defined in post-MI populations, are prevalent among cirrhotic patients and are a mortality risk factor in cirrhosis. We believe that careful assessment of autonomic function should be part of the liver transplantation evaluation, based on the high prevalence of this disorder and the risk of adverse events during hemodynamic stress.
